# A comparative assessment of three formulations of botulinum toxin A for facial rhytides: a systematic review and meta-analyses

**DOI:** 10.1186/2046-4053-2-40

**Published:** 2013-06-13

**Authors:** James P Bonaparte, David Ellis, Jason G Quinn, Mohammed T Ansari, Jessica Rabski, Shaun J Kilty

**Affiliations:** 1 Department of Otolaryngology - Head and Neck Surgery, The University of Toronto, Toronto, ON M5G 2N2, Canada; 2 Division of Facial Plastic and Reconstructive Surgery, The University of Toronto, Toronto, ON M5G 1L5, Canada; 3 Department of Otolaryngology - Head and Neck Surgery, The University of Ottawa, Ottawa, ON K1H 8L6, Canada; 4 Knowledge Synthesis Group, Centre for Practice-Changing Research, Ottawa Hospital Research Institute, Ottawa, ON K1H 8L6, Canada; 5 Art of Facial Surgery, 167 Sheppard Avenue, West Toronto, Toronto, ON M2N 1M9, Canada

**Keywords:** Botulinum toxin, Cosmetics, Skin wrinkling, Systematic review, Plastic surgery

## Abstract

**Background:**

Botulinum toxin A is a commonly used biological medication in the field of facial plastic surgery. Currently, there are three distinct formulations of botulinum toxin A, each with their purported benefits and advantages. However, there is considerable confusion as to the relative efficacy and side-effects associated with each formulation. Therefore, the purpose of this paper is to systematically assess published studies and perform a meta-analysis to determine if there is a significant advantage of any of the individual formulations.

**Methods/design:**

A systematic literature search was performed for all relevant English language randomized controlled trials using Embase, Cumulative Index to Nursing and Allied Health Literature (CINAHL), MEDLINE, World Health Organization (WHO) International Clinical Trials Registry Platform, European Union (EU) Clinical Trials Register, Cochrane Library databases of clinical trials, and ClinicalTrials.gov. Inclusion criteria included any randomized controlled trial (RCT) that assessed the use of botulinum toxin for cosmetic purposes. The included articles were also analyzed for bias using the Cochrane Collaboration’s tool for assessing the risk of bias in RCTs.

**Discussion:**

The results of this review will provide clinicians with an unbiased, high level of evidence of the comparative efficacy of individual preparations of botulinum toxin A.

**Trial registration:**

PROSPERO: CRD4201200337

## Background

Botulinum toxin type A is a commonly used biological medication for a variety of medical and cosmetic indications. Currently, there are three commonly used formulations of botulinum toxin A: onabotulinum toxin A (Botox or Vistabel (Allergen, Irvine, CA, USA), incobotulinum toxin A (Xeomin or Bocouture (Merz Pharmaceuticals, Frankfurt, Germany), and abobotulinum toxin A (Dysport (Medicis, Scottsdale, AZ, USA) or Azzalure (Ipsen, Paris, France). Each formulation is purported to have unique benefits; however, it is unclear if these differences are clinically significant. Producers of each formulation often highlight a number of factors distinguishing their product from their competitors. These features typically include: dose potency and/or equivalency [1], the onset of action [2], duration of action [3], local diffusion of the toxin [4,5], side-effect profile [6], and differences in immunogenicity.

Manufacturers of botulinum toxin typically produce their product as a 150 to 900 kDa protein. This protein includes both the primary active component (the 150 kDa polypeptide chain) [7] as well as complexing proteins. The 150 kDa chain has low activity; however, once cleaved into a 50 kDa (light chain) and a 100 kDa (heavy chain) the toxin has the ability to exert its effect [7].

There is evidence to suggest that these complexing proteins are primarily responsible for protecting the toxin as it passes through mammalian gastrointestinal tracts [8,9]. However, it has also been suggested that these proteins are responsible for the development of anti-toxin antibodies [10] which can result in the medication being ineffective. This is controversial since there is contrasting evidence, particularly given the notion that these accessory proteins may be required for adequate functioning of the active protein. For example, one of these proteins has been shown to disrupt epithelial integrity allowing the active protein entry into the cell [11].

One of the primary differences between botulinum toxin formulations is the presence or absence of these complexing proteins [1]. The manufacturers of incobotulinum toxin A use this feature in marketing to professionals, particularly with respect to the proposed low potential for antigenicity [12]. From a marketing perspective, it is understandable that manufacturers highlight this feature to distinguish it from other formulations. Clinically, however, it is unclear whether there is a significant effect of these molecular differences in terms of both antigenicity and efficacy.

With respect to dose equivalency, previous data suggest that abobotulinum toxin A and onabotulinum toxin have non-parallel dose–response curves, and thus differ in their relative potencies [1]. Studies often assess these medications at varying dose ratios with abobotulinum toxin requiring 2 to 3 times the relative dose in ‘units’ compared to the other formulations. Even though dosing is described in terms of units, it is evident that the presumed clinical effect of 1 unit is not interchangeable between formulations [13]. In addition to this, even within individual formulations, there is evidence to suggest that the specific dose influences both efficacy as well as the duration of effect, and therefore a comparison among studies is often difficult if varying doses are used [14].

More controversial, is the relative potency between onabotulinum and incobotulinum, since the dose in units is often reported as equivalent. A meta-analysis of the relative potency between these two medications was recently published [15]. The author concluded that there was no difference in relative potencies between the two products using a standard unit dosing. However, this study only assessed one outcome measure, response rate at day 30, thus limiting its usefulness. It is unclear if this method of dosing also results in similar outcomes with respect to complication rates and long-term efficacy. Furthermore, of the eight studies meeting the primary inclusion criteria of the meta-analysis, only one study included an assessment of incobotulinum toxin.

As a result of these controversies, a formal assessment of all available evidence would assist clinicians and provide a summary of the evidence. Although previous meta-analyses have been conducted, they have considerable limitations as well as narrow scopes of assessment. One previous meta-analysis assessing the duration of action of Botox did not include a formal systematic review, but rather reviewed four self-identified ‘landmark studies’ [16]. Due to this method of study selection, there is a very high risk of bias. Gadhia and Walmsley (2009) published a systematic review of botulinum toxin for facial aesthetics; however, this study only included trials comparing each medication to a placebo and excluded any active drug comparison [17]. Based on a pilot search by our team, it appeared as though this study did not identify a significant number of trials meeting their inclusion criteria. Furthermore, the authors did not undertake a formal meta-analysis of the data. Flynn (2010) conducted a review of the botulinum toxin preparations as well as their individual duration of effect [18]. This study identified a large number of peer-reviewed papers incorporating both randomized and non-randomized trials. This study identified great heterogeneity in terms of the definition of duration of action and thus comparative analysis was not attempted. Unfortunately, the inclusion of a wide range of study designs made a non-biased interpretation very difficult.

Due to the large number of non-randomized, non-blinded, industry-sponsored trials, clinicians have great difficulty determining if one specific medication carries an advantage over others in terms of both efficacy and safety. Furthermore, all previous reviews had significant flaws, not limited to poor identification of articles, a lack of a systematic review process as well as incomplete data reporting. Given the large number of double-blind randomized trials published assessing the cosmetic use of botulinum toxin, a formal systematic review and meta-analysis would assist physicians in making appropriate decisions, particularly given the significant amount of marketing by manufacturers. This review is also timely, since incobotulinum has recently been approved by Health Canada and the Food and Drug Administration (FDA) in 2013.

### Aims and objectives

The aim of this systematic review is to evaluate the comparative effectiveness and harms of three preparations of botulinum toxin A for the treatment of facial wrinkling in adult patients. When treating facial rhytides with any one of the three botulinum toxin A formulations (onabotulinum toxin A, incobotulinum toxin A and abobotulinum toxin A) in adult patients, what are the comparative treatment: 1) benefits in terms of time to treatment response, patient and/or observer assessment of rhytid reduction, and sustainability of treatment response; and 2) harms in terms of procedural complications and adverse events?

## Methods/design

This systematic review has been prospectively registered (CRD4201200337) with the PROSPERO international prospective register of systematic reviews [19]. The review will comply with the standards and guidelines proposed by the *Cochrane Handbook for Systematic Reviews of Interventions*[20]. Once completed, the reporting of our findings will adhere to the standards of the Preferred Reporting Items for Systematic Reviews and Meta-Analyses (PRISMA) [21].

### Eligibility criteria

Randomized controlled trials (RCTs) investigating the cosmetic use of a particular formulation of botulinum toxin A with no treatment, sham/placebo therapy, another formulation of botulinum toxin A, or another active treatment in adults with facial rhytides will be included. We will only include studies published in the English language. Studies which include additional cosmetic treatments in addition to botulinum toxin A during the study period (dermal fillers, skin care, or other skin therapy) will be assessed if they meet the above inclusion criteria.

### Information sources and literature search

Literature search strategies will be developed in consultation with an experienced librarian at the University of Ottawa (ON, Canada). A second librarian at the University of Toronto (ON, Canada) will perform an assessment of the search strategy and provide peer feedback. A computerized literature search will be performed independently by two reviewers and records will be de-duplicated. Articles will be identified by searching Embase (via OVID), Cumulative Index to Nursing and Allied Health Literature (CINAHL), MEDLINE, World Health Organization (WHO) International Clinical Trials Registry Platform, European Union (EU) Clinical Trials Register, Cochrane Library databases of clinical trials, and ClinicalTrials.gov (National Institutes of Health, USA). Appendix A includes the Embase and MEDLINE search strategies. Additionally, references of the retained articles will be reviewed to identify additional relevant articles that were not found with the initial search strategy. A search for other published systematic and standard reviews will be conducted to assess reference lists for missed articles. Company representatives from each of the three botulinum toxin formulations will be contacted to request unpublished trial data as well as to ensure all relevant trials have been identified.

To search the WHO International Clinical Trials Registry Platform, EU Clinical Trials Register, Cochrane Library databases and ClinicalTrials.gov, four searches will be conducted, each with one of the following keywords: botulinum toxin as well as each of the medication drug and trade names. Only clinical trials listed as completed will be included in the search. The search will be limited to articles published between the 1970 and 2013, since botulinum toxin A was not developed prior to this date.

#### Study selection process

Three reviewers will independently review record titles and abstracts to assess eligibility (JB, JQ, DE). Eligible articles will be independently reviewed by the same three reviewers in full-text form for formal inclusion in the final review. All disagreements between reviewers will be resolved during a consensus meeting. These reviewers will also assess the possibility of duplicate publications.

### Data items and data collection process

Standardized forms for data extraction will be created after pilot testing on a small subset of publications. All data will be extracted by the lead investigator (JB) and confirmed by two co-investigators (DE and JQ).

The following data will be extracted from all included studies: 1) study characteristics: author list, primary country of study, year of publication, specific type of randomized trial, duration of follow-up, number of patients randomized and participating in trial, and number and cause of drop-outs. The funding source and any other conflicts of interest will be recorded; 2) patient demographics: inclusion and exclusion criteria, age, gender, ethnicity/race, severity/classification and location of rhytides, comorbidity and co-medications, and time of previous botulinum injections (if any); 3) intervention and comparator characteristic: type of botulinum toxin A, total dose (units) and volume injected, concentration of medication, site(s) of injection and number of injections per treatment, number and frequency of treatment schedules, periprocedural precautions, injection techniques and gauge of needles used, provider training and experience (for example, physician injector versus nurse, injector patient volume per year and number of years injecting botulinum toxin), and relevant details of any other active comparator treatment; 4) outcomes: the pre-specified outcomes of interest are categorized and itemized in Table 1. Numerical outcomes data will be extracted along with their definitions, scales (for example facial wrinkle scale (FWS) [22] and glabellar line severity scale (GLSS) [23]), cut-off thresholds used for categorical data (for example, change in facial wrinkle scale of 2 required for successful treatment), and methods of assessment and monitoring.

**Table 1:** ***A priori *outcomes of botulinum toxin therapy**

***Outcomes of treatment effectiveness***	
	Time to treatment response or onset of action (based upon patient or expert/observer reported response assessment)
	Duration of action or sustainability of response
	Patient reported improvement at 30 days, and between 112 and 120 days, or nearest approximations
	Expert/observer reported improvement at 30 days, and between 112 and 120 days, or nearest approximations
	Changes in quality of life (assessed using validated instruments)
	Detection of neutralizing antibodies
***Short-term unintended effects of treatment or treatment delivery (harms)***	
	Injection site pain or hyperesthesia, edema, ecchymosis or cutaneous infections
	Urticaria
	Anaphylaxis
	Use of botulinum anti-toxin
	Headaches
	Flu-like malaise
***Longer-term unintended effects of treatment (harms)***	
	Functional neurologic deficits or disfigurements (for example brow or lid ptosis, blepharoptosis, ectropion or entropion)
	Strabismus and diplopia

### Risk of bias assessment

Using the Cochrane Collaboration’s tool for assessing risk of bias, three reviewers (JB, DE, JQ) will assess for risk of selection, performance, detection, attrition, reporting and other biases for all included studies by outcomes of interest [24]. A minimum of two reviews assessing the same risk will be required to assign a particular risk to a study characteristic. If each review scores a paper uniquely, this will be resolved at a consensus meeting. Additionally, for harms outcomes, we will assess whether outcomes were actively collected or passively measured when required.

### Data synthesis

Results will be reported descriptively first. Outcome data may be available as dichotomous, continuous, count, or time-to-event data. Relative risk for dichotomous outcomes, mean difference, ratio of means, or standardized mean differences for continuous, rate ratios of counts, and hazard ratios of time-to-event data will be preferred measures of analysis. Studies with zero events in one arm will be meta-analyzed without continuity correction with either the Peto method or the Mantel-Haenszel method [25]. Studies with zero events in both arms will be excluded from meta-analyses. When adequate head-to-head trails are available, meta-analysis will follow routine methods for pooling across studies, provided there are no major concerns about methodological and clinical diversity between studies. The random effects approach of DerSimonian and Laird will be employed for meta-analysis [26].

Indirect comparisons will be undertaken in a network meta-analysis (NMA) to investigate comparative effectiveness across the three botulinum toxins when direct evidence is deemed imprecise or absent for a given outcome. NMA is an approach to evidence synthesis which allows for the combination of direct and indirect comparative evidence of three or more treatments in a unified analysis. The applicable scenario is when treatments A and B are compared with each other when no (or few) trials of A versus B exist (that is no direct head-to-head evidence), but substantial evidence exists for trials of A versus C and B versus C exist (indirect evidence) [27-29].

It is possible that studies may have assigned interventions in trials of multiple body parts in the following two ways: 1) patients were randomized to one or another intervention, but each intervention was applied to multiple body sites and data were analyzed by body sites (outcomes data from such studies would be impacted by a clustering effect to be accounted for in our meta-analysis); and 2) each patient’s multiple body sites were randomized (the split-body design) to two different interventions and data were analyzed as if each site were a patient (outcomes data from such studies would be impacted by a crossover effect to be accounted for in our meta-analysis).

For these studies, appropriate analyses would need to factor in either the intra-class or intra-cluster correlation coefficient, or the within-patient differences and paired analysis, respectively, in addition to the between patient variability. If this is not possible, the resulting unit of analysis error may impact the precision of study summary estimates (more precise or less precise, respectively), thereby leading to inappropriate weighting in the meta-analysis [20].

Statistical heterogeneity between studies will be quantified with I-squared statistics and the *P* value from the chi-squared test (a *P* value of ≤0.10 instead of 0.05 will be used to determine statistical significance). Sparse data will not be meta-analyzed but described narratively.

Meta-regression with multiple study level covariates will be attempted when there are at least six moderate to large size studies for a continuous covariate and at least four studies for each level of a categorical covariate. Otherwise, clinical and methodological diversity in studies will be explored in subgroup analysis for the following study level covariates, data permitting [25]: 1) methodological covariates: study risk of bias and study design (trials using a split-body design versus those without or a cluster design); and 2) clinical covariates: age, sex, region of study conduct (developed versus developing countries), and prior use of toxins and procedural variability (for example expertise, precautions, needle used, dose of toxin, injection technique).

When quantitative synthesis is deemed inappropriate, a qualitative synthesis of data will be presented.

### Grading the strength of evidence and assessment of applicability

For a given outcome, reviewers’ confidence of the body of evidence in support of a conclusion will be graded as per previously published guidance [30]. Mandatory domains that will be assessed include risk of bias, consistency, directness and precision. Our pre-specified gradable outcomes are outcomes that are most likely to influence decision-making. They include: 1) patient reported outcomes (onset, efficacy at day 30 and duration of effect); 2) expert observer reported outcomes (onset, efficacy at day 30 and duration of effect); 3) local neurological defects or disfigurement; 4) headache; and 5) incidence of Botox non-responders post-therapy. For the body of evidence, we will summarize the population, intervention, comparator, setting and study duration data that may be used to assess external validity of evidence by various stakeholders and decision makers.

## Discussion

From a historical perspective, onabotulinum toxin A (Botox) was initially approved by the FDA for the treatment of strabismus and blepharospasm in 1989. In 2001, onabotulinum toxin A was approved for moderate to severe glabellar rhytides by Health Canada, followed by the FDA and EU in 2002. France, Spain and the UK soon followed in 2003, 2004 and 2006, respectively. Abobotulinum toxin A (Dysport) was not approved by the FDA for glabellar rhytides until 2009 and is still not approved by Health Canada for cosmetic indications. In 2012, a new botulinum formulation, incobotulinum toxin A (Xeomin), was approved by Health Canada for glabellar wrinkling. This product has been available in Europe and is expected to be approved for glabellar wrinkling in the USA by 2013. Given the anticipated approval by the FDA of a new medication, we feel this review will provide clinicians with the opportunity to read a non-biased, critical appraisal of the current status of the literature.

Although we are confident this review will achieve our objective, it is important to note that there are anticipated limitations and challenges. One anticipated difficulty relates to reporting and grading of outcomes, particularly efficacy outcomes. Typically, both clinicians and patients rate the extent of their wrinkling using validated scales. Commonly, the FWS is the primary outcome measure for clinician assessments, while the subject global assessment (SGA) scale is used for patient self-assessment [31]. The FWS consists of a 4-point ordinal scale ranging from no wrinkling to severe wrinkling. This scale is accompanied by a photo guide to assist in appropriate grading. The SGA is a percentage measure used to assess a change in appearance with a range from −100% to +100% improvement. Unfortunately, both these scales have been demonstrated to have low to moderate inter-rater reliability [31]. An additional scale, the GLSS [32], has demonstrated reasonable reliability measures. Similar to the FWS, this is a 4-point scale with photo guide. Interobserver reliability was noted to be 0.62, while intraobserver reliability was between 0.57 and 0.91. Furthermore, it is likely that not all studies will utilize one or both of these scales, thus complicating direct comparisons.

In addition to the actual grading method, there is likely to be variability across studies in terms of the definition of a ‘positive effect’ of treatment. The majority of studies reviewed in a pilot search defined a reduction in the FWS of 2 points as a positive effect; however, a number of studies utilized a reduction of 1 point as the definition of effect. Furthermore, some studies only included patients who started the trial with a baseline score of 2 or 3, while others included all baseline scores. To complicate issues further, facial wrinkling of patients can be assessed at rest or at maximum frown/motion, which can accentuate different areas of the face.

An additional potential difficulty will be an assessment of duration of action. Studies often utilize a variety of methods to determine and define ‘duration’. Similar to efficacy, rating scales along with a definition of response are used and followed over time until a percentage of patients no longer meet the definition of a response at a predetermined time point. However, there is no guideline as to what percentage truly represents duration. Furthermore, a major confounding variable in this regard is related to the notion that patients who have repeated treatments with botulinum toxin may have longer durations than naive patients, since there is a potential for muscle atrophy. Both factors create considerable difficulty when determining overall duration of action.

A more controversial topic is the development of anti-botulinum toxin antibodies. This study will attempt to identify cases of antibody development; however, we anticipate a number of difficulties. The evidence currently available for cosmetic botulinum toxin use suggests that although the incidence is low, there is an increased risk with repeated injections, particularly if high doses are utilized [33]. We anticipate that the majority of studies will have an inclusion criteria limiting the prior toxin use, thus there may be a considerable selection bias in terms of assessing immunogenicity. Nevertheless, we will collect this data, since it is both clinically important and highlighted as a potential distinguishing feature between toxin formulations in company marketing. Unfortunately, it is unlikely that these studies will perform formal serum antibody testing, thus reporting of antibody development is likely to be subjective and based on passive reporting. Ideally, studies should perform formal serum antibody testing to adequately identify potential non-responders. Interestingly, it is believed that if antibodies develop, switching to an alternate formulation may allow for continued treatment without the antibody targeting that specific formulation. The results of two case series suggest otherwise, since in both cases switching formulations to an additional formulation of botulinum toxin A did not result in a positive result. In two cases, patients were switched to onabotulinum, while in another two cases patients were switched to incobotulinum, both continuing to demonstrate a lack of efficacy due to antibodies [34]. We hope that this review will help to provide further evidence as well as guidance in this regard.

Even considering the potential difficulties, this review will provide clinicians and surgeons with the most up-to-date, unbiased evidence available, and will benefit patients and practitioners in the expanding field of facial cosmetic medicine.

## Appendix A

For Embase, the following strategy was used:

1) cosmetic/or dermatological agent/(20,732)

2) rhytidoplasty/or skin surgery/(7,339)

3) esthetic surgery/(10,534)

4) face surgery/or minor surgery/or plastic surgery/or skin surgery/(68,088)

5) “rhytid”.ti,ab. (51)

6) (cosmetic adj3 (procedure or injection or drug)).ti,ab. (955)

7) “frown lines”.ti,ab. (113)

8) “crow’s feet”.ti,ab. (210)

9) (muscle* adj3 (frontalis or procereus or orbicularis)).ti,ab. (2,295)

10) “wrinkle”.ti,ab. (1,119)

11) or/1-10 (101,951)

12) expbotulinum toxin A/(11,536)

13) “botox”.ti,ab. (1,781)

14) “onabotulinumtoxina”.ti,ab. (255)

15) “vistabel”.ti,ab. (17)

16) “xeomin”.ti,ab. (113)

17) “incobotulinumtoxina”.ti,ab. (41)

18) “bocouture”.ti,ab. (5)

19) “dysport”.ti,ab. (540)

20) “abobotulinumtoxina”.ti,ab. (72)

21) “azzalure”.ti,ab. (9)

22) or/12-21 (11,740)

23) 11 and 22 (968)

24) from 23 keep 1–968 (968)

For MEDLINE, the following strategy was used:

1) cosmetics/(5,232)

2) rhytidoplasty/(2,479)

3) cosmetic techniques/or rhytidoplasty/(4,807)

4) otolaryngology/or surgery, plastic/(31,779)

5) “rhytid*”.ab,ti. (1,437)

6) (cosmetic adj3 (procedure or injection or drug)).ab,ti. (737)

7) “frown lines”.ab,ti. (95)

8) “crow’s feet”.ab,ti. (157)

9) wrinkle$.ab,ti. (3,052)

10) (muscle* adj3 (frontalis or procerus or orbicularis)).ab,ti. (1,703)

11) (muscle$ adj3 “corrugator supercilii”).ab,ti. (87)

12) or/1-11 (46,436)

13) exp botulinum toxins/(11,067)

14) “botox”.ab,ti. (1,176)

15) “onabotulinumtoxina”.ab,ti. (131)

16) “vistabel”.ab,ti. (12)

17) “xeomin”.ab,ti. (51)

18) “incobotulinumtoxina”.ab,ti. (18)

19) “bocouture”.ab,ti. (6)

20) “dysport”.ab,ti. (360)

21) “abobotulinumtoxina”.ab,ti. (44)

22) “azzalure”.ab,ti. (10)

23) or/13 to 22 (11,294)

24) 12 and 23 (786)

## Abbreviations

CINAHL: Cumulative Index to Nursing and Allied Health Literature; EU: European Union; FDA: Food and Drug Administration; FWS: Facial wrinkle scale; GLSS: Glabellar line severity scale; NMA: Network meta-analysis; PRISMA: Preferred reporting for systematic reviews and meta-analyses; RCT: Randomized controlled trial; SGA: Subject global assessment; WHO: World Health Organization.

## Competing interests

DE has spoken and presented data for Allergan Inc, Canada, was on the Allergan Canada Advisory Board and is a certified trainer for Allergan Inc, Canada. The remaining authors declare that they have no competing interests.

## Authors’ contributions

JB undertook development of the research idea, project development, primary writing, manuscript preparation and literature review. DE undertook project development and manuscript preparation. JQ undertook project development and manuscript preparation. MA undertook project development, statistical analysis and data analysis protocol development. JR undertook project development, literature review and manuscript preparation. All authors read and approved the final manuscript.
